# The urgent need to move upstream in caring for people with rheumatic and musculoskeletal diseases

**DOI:** 10.1093/rap/rkac092

**Published:** 2022-11-07

**Authors:** Aicha Bouraoui, Megan Rutter, Luke Williamson, Corinne Fisher, Reecha Sofat, Debajit Sen

**Affiliations:** Department of Adolescent and Young Adult Rheumatology, University College London Hospital, London, UK; Division of Epidemiology & Public Health, Institute of Systems, Molecular and Integrative Biology (ISMIB), School of Medicine, University of Nottingham, Nottingham, UK; Department of Adolescent and Young Adult Rheumatology, University College London Hospital, London, UK; Department of Adolescent and Young Adult Rheumatology, University College London Hospital, London, UK; Department of Pharmacology and Therapeutics, University of Liverpool, Liverpool, UK; Department of Adolescent and Young Adult Rheumatology, University College London Hospital, London, UK

Coronavirus disease 2019 has exposed the significant influence of social deprivation and racial disparity on health outcomes and the urgent need to move upstream for equitable, people-centred care.

The upstream–downstream public health parable describes downstream villagers living by the side of a river, who come to the rescue of those who fall into the water upstream (Fig. 1). The older villagers recall how difficult this was initially, with scarce resources available to them. Over the years, as the downstream heroes rescued more and more people, they invested in the system. They trained teams of swimmers, bought lifeboats and even built a hospital by the side of the river. In fact, they were so busy that they did not have the time to go upstream and understand why people were falling into the river in the first place [1].

**Figure 1.**
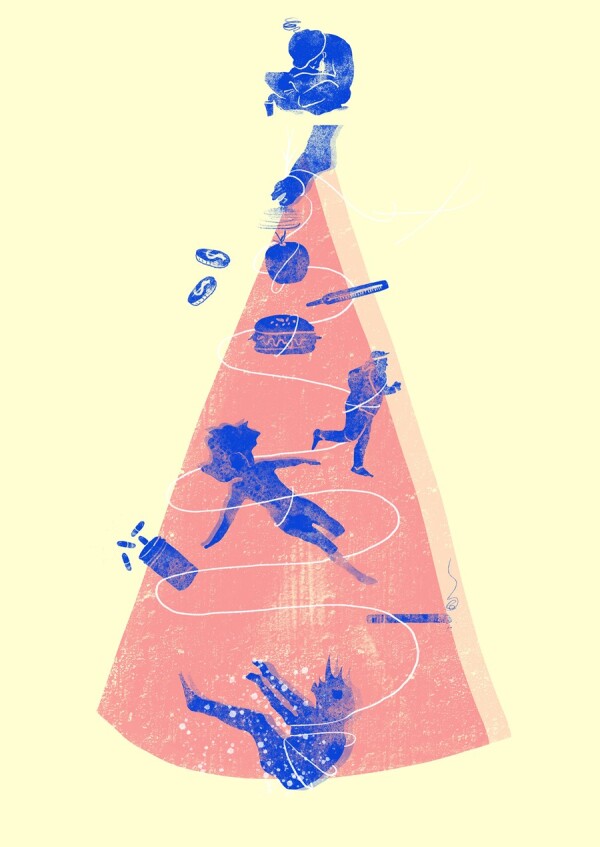
The upstream–downstream public health parable

With inequality ever more exposed, the challenge to us as a health-care community is to move away from our downstream, siloed working to more preventive upstream approaches. We need a system underpinned by population health measures to improve quality and efficiency.

A recent Versus Arthritis report revealed that one-third of the population in England have some degree of chronic pain (CP) and >5 million suffer from high-impact CP (defined as CP sufficiently severe that people are unable to carry out their daily activities) [2]. Notably, the prevalence of high-impact CP in the most deprived areas is 30%, compared with 15% in the least deprived. High-impact CP was also more prevalent amongst Black (45%) and Asian (35%) ethnicities, who are also more likely to experience unemployment, financial insecurity and exposure to adversity and racism, all of which can impact pain outcomes [2–4]. There has also been a concerning rise of high-impact CP in young people, from 21% in 2011 to 32% in 2017. Plausible explanations include reduced physical activity, the rising prevalence of obesity and adverse socioeconomic circumstances [2].

In the UK, analysis from the National Early Inflammatory Arthritis Audit revealed that Black, Asian and minority ethnic groups are less likely to achieve remission from inflammatory arthritis compared with White ethnic groups [5]. Similar findings were noted in The Netherlands, where inflammatory arthritis patients from low socio-economic backgrounds experienced adverse disease outcomes [6].

Our current treat-to-target pathways focus on earlier diagnosis and medical intervention to improve outcomes for people with inflammatory arthritis. Yet, despite advances in treatment, up to two-thirds of patients with inflammatory arthritis report that their pain is not well controlled [7]. Pain, a complex sensory, emotional and cognitive experience, is influenced by several elements, including social and cultural factors. Although the treat-to-target approach plays a key role in improving DASs, it ignores major disease sequelae, including fatigue, low mood and impact on activities of daily living.

The term syndemic describes ‘the presence of two or more disease states that adversely interact with each other’ [8], a situation more commonly seen in the presence of negative social circumstances [8]. Identifying and addressing co-morbidities and their societal drivers is vital if we are to improve the symptoms that matter to patients. In fact, in long-term conditions, such as inflammatory arthritis, medical interventions account for only 10–20% of the factors that impact outcomes [9]. Social determinants of health, including socioeconomic, cultural and environmental factors and health-related behaviours, account for 80–90% [9, 10]. To improve outcomes, we need to look upstream to understand the drivers of poor health, to identify the most vulnerable and to design prevention strategies tailored to those groups.

An upstream approach should involve a deeper understanding of the patterns and generators leading to adverse health outcomes. We need to move away from our current reactive, demand-led approach to a proactive, holistic and leaner one, aiming to prevent chronic diseases, reduce their complications and save lives.

Such a strategy is challenging to implement, and for it to work well it must be underpinned by strong partnerships and system integration. Although primary care has a crucial role in prevention, it is the responsibility of the whole system collectively to shift from a reactive to a proactive approach, integrating the social determinants of health into clinical pathways.

What is the role of the rheumatology community in driving high-quality, equitable care for people with rheumatic diseases? And how can we embed population health in our clinical pathways?

Firstly, we need to improve people’s understanding and raise awareness of the factors that influence health outcomes beyond the biological aspects of disease. This can be delivered in the form of workshops and collaborative meetings to develop a shared vision and collective understanding.

Secondly, we need high-quality data to identify variations within and between health systems. We need to question why certain problems persist. One strategy is the ‘5 whys’, a technique supported by the World Health Organization [11], in which one asks iteratively why a problem exists until the root cause is reached. Why are Black, Asian and minority ethnic patients less likely to achieve remission? Why do they experience more anxiety and depression compared with White individuals [5]?

As yet, there is limited research investigating the impact of interventions on social determinants of CP, and it has been recommended by the National Institute for Health and Care Excellence as an area of future research [12]. Along with high-quality data, we need robust analytics to, for example, identify groups at risk of disease complications, who might benefit the most from intensive, targeted resources and support.

Thirdly, we must collaborate with stakeholders to influence broader social policies beyond the health-care system and take a preventative approach. Reaching out to other sectors, such as education and employment, is key; for example, the promotion of wellness through physical activities and health and wellness schemes in schools and workplaces [12, 13].

Finally, the involvement of people with lived experience of rheumatic disease is integral to implementing effective pathways. Local populations should be involved in co-designing services, identifying vulnerable groups and mobilizing resources, including community assets [14].

Although there has been significant improvement in the care of people with rheumatic diseases, there is increasing evidence of growing inequality and social disadvantage entrenching poor health outcomes from the earliest ages [3]. Addressing the interplay between social determinants of health and rheumatic diseases demands a broader policy focus, alongside innovative medical interventions. We need urgently to move upstream, beyond our siloed health-care systems, to provide equitable, person-centred care for all.

## Data Availability

No new data are presented in this paper.
